# Changes in weight status during the COVID-19 pandemic: impact of educational level and mental health

**DOI:** 10.1093/eurpub/ckad188

**Published:** 2023-11-15

**Authors:** Siri Rosenkilde, Thorkild I A Sørensen, Maria H Algren, Lau C Thygesen

**Affiliations:** National Institute of Public Health, University of Southern Denmark, Copenhagen K, Denmark; National Institute of Public Health, University of Southern Denmark, Copenhagen K, Denmark; Department of Public Health, Novo Nordisk Foundation Center for Basic Metabolic Research, Faculty of Health and Medical Sciences, University of Copenhagen, Copenhagen K, Denmark; National Institute of Public Health, University of Southern Denmark, Copenhagen K, Denmark; National Institute of Public Health, University of Southern Denmark, Copenhagen K, Denmark

## Abstract

**Background:**

The COVID-19 pandemic has resulted in a disruption of daily routines and changes in health behaviors leading to widespread concerns about unfavorable changes in weight status and a potential increase in the prevalence of obesity. This study examined the long-term impact of the COVID-19 pandemic on changes in weight status and its possible dependency on educational level and mental health.

**Methods:**

The study utilizes the Danish Health and Well-being Survey with repeated self-reported information on weight status collected before the COVID-19 pandemic (autumn of 2019) and twice during the pandemic (autumns of 2020 and 2021). Information on educational level was derived from registers, whereas mental health was measured using validated scales. Generalized estimating equations were performed to investigate changes in mean weight and body mass index (BMI) category (BMI < 30 to BMI ≥ 30) between 2019 and 2021 and to investigate potential differences in changes in weight status by pre-pandemic educational level and mental health.

**Results:**

Mean weight significantly increased by 0.34 kg [95% confidence interval (CI): 0.16–0.51] in 2020 and by 0.46 kg (95% CI: 0.26–0.66) in 2021 compared with pre-pandemic weight status. The increase was greater among individuals with lower educational levels and poorer mental health. There were no significant changes in BMI category during the pandemic.

**Conclusion:**

The results showed a significant increase in mean weight among the Danish population, particularly among individuals with lower educational levels and poorer mental health, but without detectable differences in obesity, supporting a long-term but minor impact of the COVID-19 pandemic on weight status.

## Introduction

In March 2020, the World Health Organization (WHO) declared the coronavirus (COVID-19) outbreak a global pandemic.^1^ Consequently, governments worldwide established lockdown measures to prevent further disease transmission, forcing people to stay at home and maintain social distancing for long periods of time. In Denmark, such lockdown measures came into effect in March 2020.^2^ The situation resulted in a disruption of daily routines (e.g. social distancing, working from home, and lockdown of several functions of society) along with enhanced psychosocial uncertainty. As a result, changes in health behaviors have repeatedly been reported^3–5^ leading to widespread concerns about unfavorable weight changes and a potential increase in the prevalence of obesity and its metabolic comorbidities.^6^^,^^7^

The COVID-19 pandemic is likely to have had both short- and long-term negative effects on mental health,^8–10^ which might induce weight gain. A study investigating the association between the COVID-19 pandemic and weight gain reported a higher increase in weight among those with anxiety, depression or symptoms of both.^11^ Furthermore, symptoms of anxiety, depression and stress have been associated with a negative impact on dietary behaviors^12^^,^^13^ and physical inactivity.^14^ A study among 1855 adults demonstrated that participants who were not physically active before and during the lockdown had the highest level of depression and gained more weight during the lockdown period compared with physically active participants.^15^

Lockdown measures related to the COVID-19 pandemic might further result in a worsening in socioeconomic conditions such as financial and job loss. Socioeconomic challenges most often affect the more disadvantaged people in society and thereby exacerbate socioeconomic inequality.^7^ Unfortunately, a potential widening in societal inequality might induce an increase in overweight in groups with a lower socioeconomic status, as these groups tend to have a higher prevalence of health risk behaviors compared with higher socioeconomic groups.^16^^,^^17^

As obesity and consequent comorbidities are major public health concerns, investigating changes in weight status possibly imposed by the COVID-19 pandemic and subsequent lockdown measures is crucial to better understand the long-term health consequences of the pandemic. Using a Danish representative longitudinal study with repeated self-reported data on weight changes measured just before the COVID-19 pandemic and twice during the pandemic, we investigated (i) changes in weight status from before the COVID-19 pandemic through 2020 and 2021 and (ii) its possible dependency on educational level and mental health.

## Methods

### Study population

This study utilizes the Danish Health and Well-being Surveys collected in 2019, 2020 and 2021 among the same individuals.^18^ The Danish Health and Well-being Surveys cover the following four topics: health status (including self-rated health, chronic conditions, and mental health), health determinants (including weight and height, smoking status and alcohol consumption), healthcare utilization (use of healthcare services) and socio-demographic characteristics (including sex, age and educational level).

In the first survey, 14 000 individuals aged 15 or older were randomly selected from the Danish Civil Registration System^19^ and were invited to participate in the survey. In total, 6629 individuals (response rate 47%) participated in the survey. In the second survey, the same individuals who still were alive, resident in Denmark, and who had not declined to participate in the first survey, were reinvited to participate (*n* = 13 474). A total of 6712 individuals (response rate 50%) participated in the second survey. In the third survey, the same approach was used (*n* = 13 182). In addition, 388 individuals aged 15–16 years were invited to participate in the third survey to ensure a random selection among adults over the age of 15 years. In total, 5384 (response rate 40%) participated in the third survey.

The first survey was conducted in September–December 2019 (before the COVID-19 outbreak). The second survey was conducted in September–November 2020 during the COVID-19 pandemic. By August 2020, the number of COVID-19 cases in Denmark increased, and several restrictions were applied including a limitation of social gatherings of more than 10–50 people; closing of restaurants, bars and cafés no later than 10 p.m.; general request to work from home; social distancing; and the use of face masks in public indoor areas. By the time the third survey was conducted in September–December 2021, COVID-19 was no longer considered a critical societal threat and most restrictions were removed.^20^

### Measures

#### Outcome measure

The primary outcome of interest was changes in mean weight. The secondary outcome was changes in body mass index (BMI) category from BMI <30.0 to BMI ≥30.0. Participants self-reported their weight and height. BMI was calculated as weight (kg) divided by the square of height (m^2^).

#### Explanatory variables

*Educational level*: Information on educational level was derived from Statistics Denmark’s population-based registers^21^ and was categorized into elementary school, upper secondary/vocational education and higher education.

*Mental health*: The Short Warwick-Edinburgh Mental Wellbeing Scale is a measure of mental well-being and consists of seven items, each with five response categories. The score ranges from 7 to 35 where higher scores indicate greater mental well-being.^22^ In this study, raw scores were converted into metric scores to enhance scaling properties and to allow comparisons among different studies.^22^^,^^23^ Scores of 19.98 or less represent probable depression; sores between 20.73 and 28.13 represent average mental well-being; and scores of 29.31 or more represent high mental well-being.^24^ The eight-item Patient Health Questionnaire depression scale (PHQ-8) is a measure of depression with a score ranging from 0 to 24 with higher scores indicating greater depression.^25^ As recommended, PHQ-8 was included as a binary variable with a score of 10 or more representing probable depression.^25^

### Statistical analyses

Descriptive statistics were used to describe the study sample. Percentages, mean weight in kilograms and their corresponding standard deviations (SDs) were calculated for all variables.

Generalized estimating equation (GEE) models were used to investigate changes in weight status from before the COVID-19 pandemic (autumn of 2019) and twice during the pandemic (autumns of 2020 and 2021). GEE was applied to take account of the correlation between repeated observations and the ability to handle missing data.^26^

First, a linear GEE model including time as the predictor variable was performed to estimate the differences in mean weight from 2019 through 2020 and 2021 for the full sample. Subsequently, three models were performed to assess differences in mean weight over time by educational level and mental health (mental well-being and depression) measured in 2019. Thus, added interaction terms between time and educational level, mental well-being and depression were included in respective models. All models were adjusted for age, sex, ethnicity and height. The models including mental health were further adjusted for educational level. Wald statistics were applied to test for statistical significance using a significance level of 0.05.

Second, a logistic GEE model including time as the predictor variable was performed to estimate the odds of BMI category change (from BMI < 30 to BMI ≥ 30) from 2019 through 2020 and 2021 for the full sample. Subsequently three models were performed to assess differences in odds of BMI category change over time by educational level and mental health using the same approach as described above. All models were adjusted for age, sex and ethnicity. The models including mental health were further adjusted for educational level.

All GEE models were specified with an unstructured working correlation. All analyses were conducted using STATA version 16.0 on the research platform provided by Statistics Denmark.

#### Weighting

All descriptive (except numbers and percentages) and analytical analyses were weighted to account for non-response bias in the three survey years. When calculating weights, we estimated the probability of participating and responding to questions on weight and height among those invited by age in 5-year categories and sex.

### Ethical aspects

Participation in the surveys was voluntary. In Denmark, register and questionnaire studies do not require approval by committees on biomedical research ethics according to Danish legislation. The surveys were approved by SDU Research & Innovation Organization (RIO).

## Results

A total of 8208 individuals reported information on weight and height. Of these, 2742 persons participated once, 2296 persons participated twice, and 3170 persons participated in all three surveys. After the exclusion of missing information on ethnicity (*n* = 51), a total of 16 793 observations were eligible for analyses.

### Baseline characteristics of study participants in 2019

Baseline characteristics of study participants in 2019 are shown in table 1. The study population in 2019 consisted of 6224 participants with a mean weight of 77.8 kg (SD 17.4). In all, 16.7% of the participants reported low mental well-being, 8.0% reported signs of depression and 16.8% had a BMI ≥30. Mean weight was higher among men, participants between the ages of 45 and 59 years, participants with upper secondary or vocational education, and participants with signs of depression.

**Table 1:** Baseline characteristics of study participants in 2019 and mean weight (weighted for non-response)

	Total	%	Mean weight (SD)
All	6224	100.0	77.8 (17.4)
Sex			
Men	2685	43.1	85.5 (16.5)
Women	3539	56.9	70.6 (15.2)
Age (years)			
15–29	876	14.1	72.7 (16.7)
30–44	1036	16.7	79.3 (18.6)
45–59	1706	27.4	81.5 (17.4)
60–74	1825	29.3	79.1 (16.7)
≥75	781	12.6	73.1 (14.5)
Educational level			
Elementary school	1403	22.5	75.4 (18.0)
Upper secondary education/vocational education	2544	40.9	80.3 (17.8)
Higher education	2277	36.6	76.7 (16.3)
Ethnicity			
Danish	5780	92.0	78.1 (17.4)
Immigrants/descendants	444	8.0	73.6 (17.3)
SWEMWBS			
Low	983	16.7	77.9 (19.3)
Average	3527	60.0	77.9 (17.4)
High	1371	23.3	77.6 (15.9)
Depression (PHQ-8)			
No depression	5492	92.0	77.7 (17.0)
Depression	478	8.0	78.6 (21.7)
Obesity			
BMI <30	5178	83.2	73.0 (12.6)
BMI ≥30	1046	16.8	103.0 (17.9)

PHQ-8, The eight-item Patient Health Questionnaire Depression Scale; SWEMWBS, Short Warwick-Edinburgh Mental Wellbeing Scale.

### Changes in weight status of study participants

The mean weight of study participants significantly increased by 0.34 kg [95% confidence interval (CI): 0.16–0.51] in 2020 and by 0.46 kg (95% CI: 0.26–0.66) in 2021 compared with pre-pandemic weight status, when adjusting for age, sex, ethnicity and height (table 2, model 1). Furthermore, educational level was significantly associated with mean weight. Higher mean weight was observed among participants with upper secondary or vocational education (mean difference 2.69, 95% CI: 1.72–3.66) and lower mean weight was observed among participants with higher education (−1.08, 95% CI: −2.05 to 1.10) compared with participants with elementary school (table 1, model 2). In addition, mental well-being and depression were significantly associated with mean weight. The highest mean weight was observed among participants with low mental well-being (2.60, 95% CI: 1.20–4.00) and depression (3.78, 95% CI: 1.90–5.66) (table 2, models 3 and 4).

**Table 2:** Generalized estimating equations model coefficients on weight status from 2019 through 2020 and 2021 (weighted for non-response)

	Mean difference in kg (95% CI)	OR of BMI ≥ 30 (95% CI)
Model 1 (*n* = 16 793)		
Time		
2019	0.00	1.00
2020	0.34 (0.16 to 0.51)	1.05 (1.01 to 1.10)
2021	0.46 (0.26 to 0.66)	1.05 (1.00 to 1.11)
Model 2 (*n* = 16 793)		
Educational level		
Elementary school	0.00	1.00
Upper secondary education/vocational education	2.69 (1.72 to 3.66)	1.02 (0.87 to 1.20)
Higher education	−1.08 (−2.05 to −0.10)	0.52 (0.44 to 0.63)
Model 3 (*n* = 13 306)		
Mental well-being (SWEMWBS)		
High	0.00	1.00
Average	0.67 (−0.25 to 1.58)	1.08 (0.91 to 1.29)
Low	2.60 (1.20 to 4.00)	1.47 (1.18 to 1.84)
Model 4 (*n* = 13 306)		
Depression (PHQ8)		
No depression	0.00	1.00
Depression	3.78 (1.90 to 5.66)	1.73 (1.38 to 2.18)

Notes: All models including weight as the outcome are adjusted for age, sex, ethnicity and height. All models including BMI as the outcome are adjusted for age, sex and ethnicity. Models 3 and 4 are further adjusted for educational level. PHQ-8, The eight-item Patient Health Questionnaire Depression Scale; SWEMWBS, Short Warwick-Edinburgh Mental Wellbeing Scale.

No significant differences in the odds of changing BMI category from BMI <30 to BMI ≥30 were observed in 2020 or 2021 compared with 2019 (table 2, model 1). Participants with higher education had significantly lower odds of a BMI ≥30 compared with participants with elementary school [odds ratio (OR) 0.52, 95% CI: 0.44–0.63] (table 2, model 2). In addition, significantly higher odds of a BMI ≥30 were observed among participants with low mental well-being (OR 1.47, 95% CI: 1.18–1.84) and depression (OR 1.73, 95% CI: 1.38–2.18) compared to participants with high mental well-being and no depression (table 2, models 3 and 4).

### Educational level, mental health and mean weight

**Figure 1:**
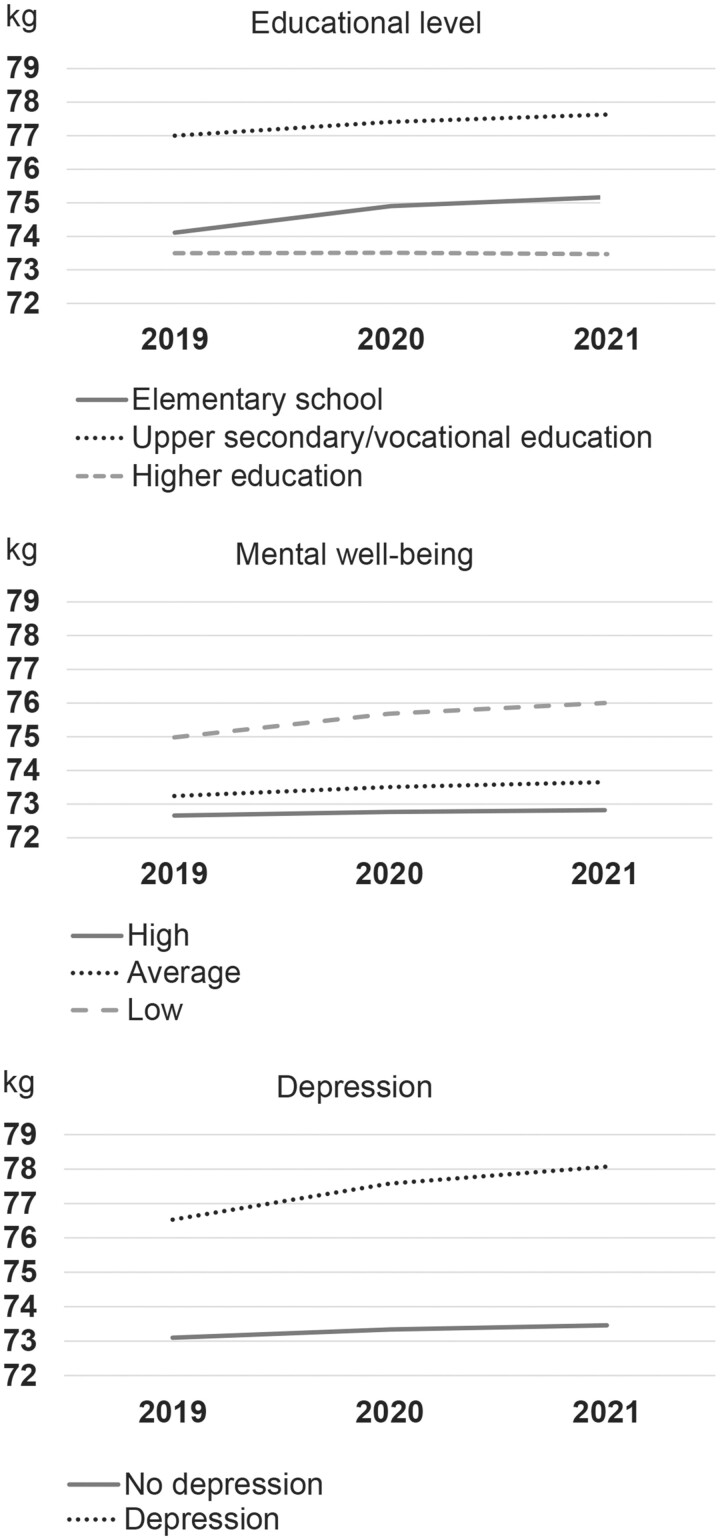
Interaction between educational level, mental well-being, depression, respectively, and time on mean weight in 2019, 2020 and 2021. Exemplified with a female, aged 45–59, height of 170 cm and with elementary education

To investigate whether educational level modified changes in mean weight from 2019 through 2020 and 2021, an interaction term between educational level and time was added to the main model and was found to be significant (*P = *0.003). The interaction between educational level and time on mean weight is illustrated in figure 1. The mean weight in figure 1 is exemplified with a female, aged 45–59 years, and with a height of 170.0 cm. The highest increase in mean weight from 2019 and through 2020 and 2021 was observed among participants with elementary education, and a minor increase in mean weight was observed among participants with upper secondary or vocational education. Thus, the difference in mean weight among participants with elementary education and upper secondary or vocational education was smaller in 2021 compared with pre-pandemic weight status. Participants with higher education had a stable weight across the three time points.

The interaction between mental well-being, depression and time on mean weight is also illustrated in figure 1 exemplified with a female, aged 45–59 years, height 170.0 cm and with elementary school. Although a greater increase in mean weight from 2019 through 2020 and 2021 was observed among participants with low mental well-being and depression compared with participants with higher mental well-being and no depression, the differences in development over time were not significant (*P* = 0.24 for mental well-being and *P* = 0.16 for depression).

### Educational level, mental health and changes in BMI category

**Figure 2:**
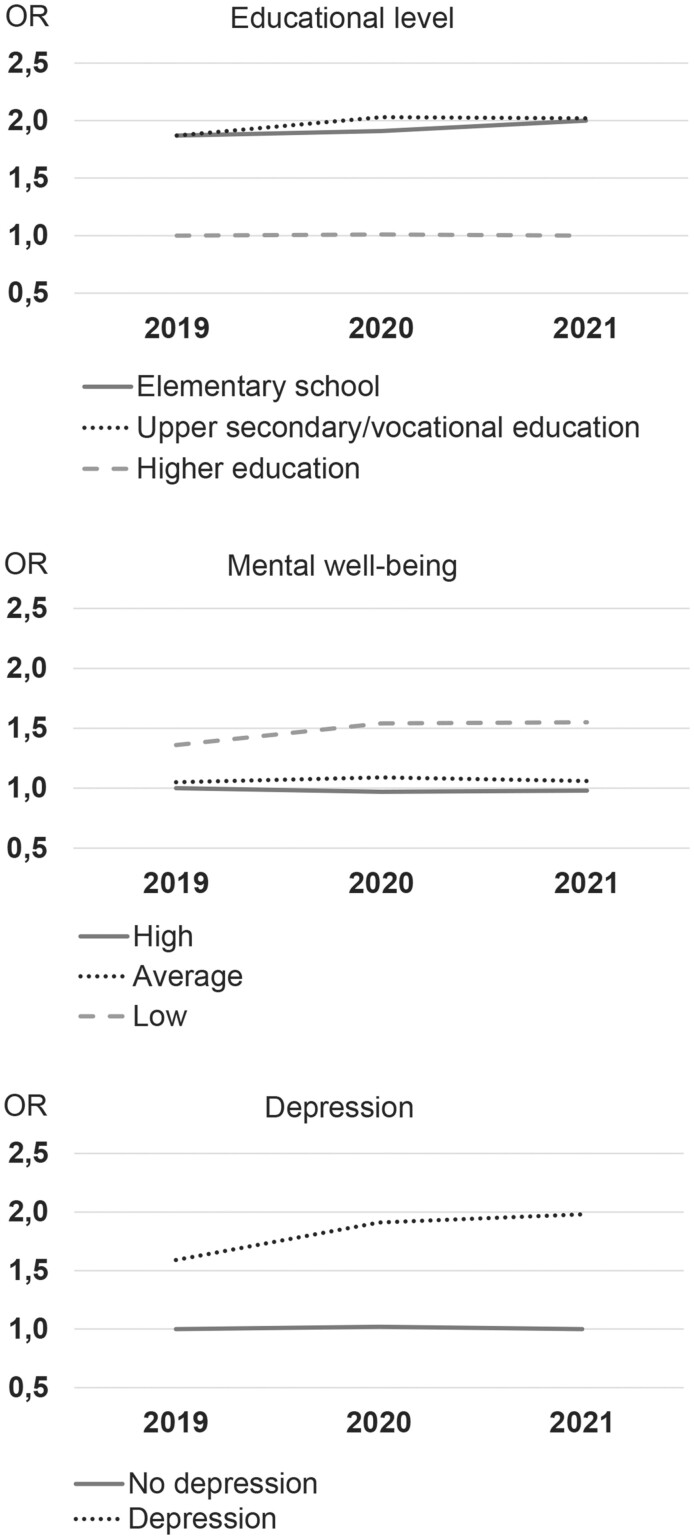
Interaction between educational level, mental well-being, depression, respectively, and time on odds of changing BMI category in 2019, 2020 and 2021. Exemplified with a female, aged 45–59, height 170.0 cm and with elementary education

To investigate whether educational level modifies the odds of changing BMI category from 2019 through 2020 and 2021, an interaction term between educational level and time was added to the main model. The interaction between educational level and time on BMI category change is illustrated in figure 2 and is exemplified with a woman, aged 45–59 years, and with a height of 170.0 cm. A greater increase in odds of changing BMI category from 2019 through 2020 and 2021 was observed among participants with upper secondary or vocational education compared to participants with higher educational levels. A similar, although slightly smaller, increase was observed among participants with elementary education. However, the interaction was not significant (*P = *0.6013).

The interaction between mental well-being, depression and time on BMI category change is also illustrated in figure 2 and is exemplified with a woman, aged 45–59 years, height 170.0 cm and with elementary school. A greater increase in odds of changing BMI category was observed among participants with low mental well-being and depression from 2019 to 2020 compared with participants with high mental well-being and no depression. From 2020 to 2021 the odds were stable in both groups. No significant interaction was observed (*P *=* *0.4036 for mental well-being and *P *=* *0.1043 for depression).

## Discussion

This study aimed to investigate changes in weight status among the Danish population in a longitudinal study comparing weight status measured just before the outbreak of the COVID-19 pandemic and twice during the pandemic; during autumn 2020 when the spread of the virus increased and during autumn 2021 when the spread of the virus declined. Mean weight significantly increased by 0.34 kg in autumn 2020 and by 0.46 kg in autumn 2021 compared with pre-pandemic weight status. Our results demonstrated inequalities in weight status by educational level and mental health status before the COVID-19 pandemic and that these inequalities have been exacerbated during the COVID-19 pandemic. During the pandemic, the increase in mean weight was significantly greater among participants with elementary education and upper secondary or vocational education compared with participants with higher education. Although a greater increase in mean weight was observed among participants with low mental well-being and depression, the differences were not significant. There were no significant changes in BMI category from 2019 through 2020 and 2021, and the association was not modified by either pre-pandemic educational level or mental health status.

Previous studies have consistently investigated the short-term impact of the COVID-19 pandemic on changes in weight, although not taking pre-pandemic weight trends into consideration. A combined systematic review and meta-analysis reported a significant increase in self-reported weight by 1.57 kg during the first lockdown period (March–May 2020) compared with before the pandemic.^27^ A longitudinal study among US adults demonstrated a significant increase in mean weight by 0.62 kg during the ‘peak-lockdown’ period (April–May 2020) and the ‘post-lockdown’ period (September–October 2020).^3^ Another longitudinal study among UK adults similarly reported a significant increase in mean weight between May–June 2020 and August–September 2020 (74.95–75.33 kg) followed by a significant decrease in November–December 2020 (75.06 kg) compared with May–June levels.^4^

Few studies have investigated the long-term impact of the COVID-19 pandemic on weight changes.^28^^,^^29^ A longitudinal study investigated changes in mean weight from before 2020 (years 2018 and 2019) to 2021 and demonstrated a significant weight gain of 0.33 kg without taking pre-pandemic weight trends into consideration,^28^ which is comparable to our findings. In addition, a study among 4.25 million US adults followed from January 2019 through May 2021 showed a small increase (0.1 kg) in weight during the first year of the pandemic (March 2020 through March 2021) when taking pre-pandemic weight trends into consideration.^29^ Thus, these findings are in line with our findings on the long-term impact of the COVID-19 pandemic on changes in weight status into late 2020 and 2021 compared with pre-pandemic weight status.

Our results also support subgroup differences in changes in weight. We observed a greater increase in weight among participants with lower educational levels compared to participants with higher educational levels. This finding might be due to the vulnerability often associated with individuals in these groups, including scarce financial resources, unfavorable health behaviors and high levels of morbidity.^30^^,^^31^ However, previous studies have shown contradictory results, as some studies suggest that educational level is associated with COVID-19 weight gain,^11^^,^^32^ while other studies have found no such association.^5^^,^^33^

We also observed subgroup differences in changes in weight among participants with different levels of pre-pandemic mental health, and particularly in regard to depression. Although the results were not significant, we observed a greater increase in mean weight among participants with low mental well-being and depression, which is in line with previous studies suggesting that individuals with poor mental health are more likely to gain weight.^11^^,^^34^ A population-based study among US adults reported that participants with anxiety, depression or symptoms of both were more likely to report weight gain than participants without these symptoms.^11^ Similarly, a longitudinal US study investigating the effects of stay-at-home mandates on weight status between May and August 2020 also reported a greater weight gain among participants with depressive symptoms than among those without depressive symptoms.^34^ The finding of the present study could be explained by the fact that feelings of lower mental well-being and depression may not be constant over time. Contrary to previous studies, the follow-up time in the present study is much longer (1 year between each measurement). Thus, some of the study participants experiencing lower mental well-being and depression in 2019 may not have these feelings at follow-up in 2020 and 2021.

The observed weight gain in this study must be compared with the expected weight changes based on pre-pandemic weight trends. In Denmark, men and women have had an average weight gain of 1.5 and 1.6 kg in the period between 2010 and 2017, corresponding to an annual weight gain of 0.21 and 0.23 kg, respectively.^35^ In addition, weight gain during the holiday season has been described as an important contributor to the annual weight gain.^36^ A narrative review investigating the effect of the holiday season on weight gain reported a significant weight gain of 0.4–0.9 kg during the holiday season from November to January,^37^ which is similar to the findings of this study. Thus, despite that the COVID-19 pandemic undoubtedly has had a marked impact on everyday life and health behavior patterns, the observed weight gain in this study is only slightly higher compared with the expected annual weight gain and the weight gain observed during the holidays.

We also investigated changes in BMI category during the COVID-19 pandemic, but no significant changes from BMI <30 to BMI ≥30 were observed. A longitudinal study investigating changes in BMI category among 165 279 Saudi Arabians found that 4.8% of the normal weight participants became overweight or obese, while 5.1% of the participants changed from being overweight to obese. The authors conclude that the increase in the prevalence of overweight and obesity will contribute to the existing public health burden of obesity in Saudi Arabia.^28^ However, no increase in the prevalence of overweight and obesity was found in this study underlining the fact that, although a small increase in mean weight was observed, the changes in weight status observed in this study do not represent a major public health concern. However, previous research has emphasized that even small changes in weight status can become permanent and lead to substantial weight gain over time potentially contributing to an increased prevalence of overweight and obesity.^38^

This study has several strengths, including a rather large national sample and the longitudinal study design with repeated measures of weight status. Furthermore, the prospective collection of data and the measure of weight status just before the COVID-19 pandemic (reflecting a true baseline measurement) are considered strengths of this study. Thus, this study builds and expands upon previously published studies by providing a greater understanding of the long-term impact of the COVID-19 pandemic on changes in weight status. Some limitations are also present. Information on weight was self-reported and may thus be underestimated.^39^ However, since we are interested in weight changes over time and not in the actual weight of the participants, we do not believe this affects the results of this study. Finally, our study may be influenced by selection bias since the response proportions were rather low. To limit the influence of selection bias, we weighted the analyses by response weights based on the age and sex distributions of the invited sample. However, selection bias might still be present, as individuals with overweight or obesity, who are more susceptible to weight gain, are known to have lower response proportions in health surveys.^40^

In this sample of Danish adults, mean weight significantly increased by 0.34 kg in 2020 and by 0.46 kg in 2021 compared with pre-pandemic weight status, but no significant changes in BMI category were observed. The increase was significantly greater among participants with lower educational levels compared with participants with higher educational levels and among participants with low mental well-being and depression, although the results were not significant. Thus, our results support a long-term but minor impact of the COVID-19 pandemic on weight status.

## Data Availability

Data are linked to administrative registers and can only be accessed through affiliation with University of Southern Denmark. The authors welcome any contacts regarding collaboration. The questionnaires (in Danish) are accessible at www.sdu.dk/da/sif/forskning/projekter/betydningen_af_covid_19_krisen.
